# A comprehensive evaluation for polymorphisms in *let-7* family in cancer risk and prognosis: a system review and meta-analysis

**DOI:** 10.1042/BSR20180273

**Published:** 2018-07-31

**Authors:** Ben-Gang Wang, Li-Yue Jiang, Qian Xu

**Affiliations:** 1 Department 1 of General Surgery, the First Hospital of China Medical University, Shenyang 110001, Liaoning Province, China; 2 The Institute of General Surgery, the First Hospital of China Medical University, Shenyang 110001, Liaoning Province, China; 3 The Clinical Medicine, The Fourth Military Medical University, Xi’an 710000, Shanxi Province, China

**Keywords:** cancer, Let-7, meta-analysis, single nucleotide polymorphism, system review

## Abstract

miRNA polymorphisms had potential to be biomarkers for cancer susceptibility and prognosis. The mature *miRNA-let-7* family was considered as the most important miRNA for the cancer incidence and progression. Recently, the promising *let-7* miRNAs were reported to be associated with various cancers, but the results were inconsistent. We performed a first-reported systematic review with a meta-analysis for the association of *let-7* family single nucleotide polymorphisms (SNPs) with cancer risk/prognosis. Ten studies were included with a total of 3878 cancer cases and 4725 controls for the risk study and 1665 cancer patients for the prognosis study in this meta-analysis. In the risk study, the *let-7i* rs10877887 and *let-7a-1/let-7f-1/let-7d* rs13293512 were shown no significant association for the overall cancer risk. In the stratified analysis, the rs10877887 variant genotype was significantly associated with a decreased cancer risk in head and neck cancer (TC compared with TT: *P*=0.017; odds ratio (OR) = 0.81; TC + CC compared with TT: *P*=0.020; OR = 0.82). In the prognosis study, the let-7i rs10877887 SNP was shown to be associated with a higher risk for cancer prognosis in the dominate model (*P*=0.004; hazard ratio (HR) = 1.32). The other two SNPs (*let-7a-1* rs10739971 and *let-7a-2* rs629367) were not found to be associated with cancer survival. None of the *let-7* family polymorphisms had potential to be biomarkers for cancer susceptibility but *let-7i* rs10877887 SNP had potential to be predicting markers for cancer prognosis. In the future, large-sample studies are still needed to verify our findings.

## Introduction

From this century, miRNAs were considered as star molecules, instead of ‘trash’, as they worked as a regulatory element for the post-translation of mRNA [1]. miRNAs were also generated from the genome DNA and could transcript and translate into mature miRNA, which was executed in two steps: from pri-miRNA to pre-miRNA, and from pre-miRNA to mature miRNA [2]. As miRNA is small (19–24 nt long) [3], it has the characteristic of stability and thus, has the potential to be the biomarker for the detection in tissues, or even in serum or urine [4]. Other characteristics of miRNA are: first, it could complementarily combine with multiple target sequences and one miRNA could regulate multiple different target genes [1]; second, it has little chance to vary or to mutate [5]. But, if there is a variation in the formation process of miRNA, it could affect the quality and quantity of mature miRNA and even affect hundreds of targeted genes regulated by the changed miRNA [6].

Single nucleotide polymorphisms (SNPs) are the common variation in the genetic polymorphisms and are known as the potential biomarkers for the forecast in cancer risk and predicting the cancer prognosis [7]. Pri-miRNA and pre-miRNA have SNPs which were studied to be associated with cancer risk and prognosis [8,9]. As pri-miRNA is always 500–3000 bp long and pre-miRNA is 60–70 bp long, the existence of pre-miRNA SNPs is limited, and pri-miRNA SNPs are more relative and reported to affect the function of miRNAs [5].

*Let-7* family is one of the earliest found miRNAs and composed of ten kinds of miRNAs (*let-7a, let-7b, let-7c, let-7d, let-7e, let-7f, let-7g, let-7i, miR-98*, and *miR-202*) [10]. *Let-7* family is the most important miRNA acting on carcinogenesis, as Krol et al. found, the pri-miRNA of *let-7* family could combine with LIN28 and suppress the splicing procedure of Drosha and Dicer, two important restriction enzymes involved in the maturation process for all miRNAs [11]. In addition, by knocking down the Drosha enzyme to suppress all the miRNA maturation processes comprehensively, Kumar et al. found that the main reason for the activation and promotion of cell’s malignant transformation was the downregulation of *let-7* family expression [12]. Thus, *let-7* family is essential to suppress the cancer cells’ proliferation, and plays important roles in the carcinogenesis process [13]. The *let-7* genetic polymorphisms could have participated in the carcinogenesis process.

The *let-7* genetic polymorphisms were reported to be associated with cancer risk and prognosis, but the results were inconsistent. For example, Jing Liu et al. found the *let-7i* promoter rs10877887 SNP variant C allele could increase cancer risk (odds ratio (OR) = 1.35) [14] while others found the variant C allele could decrease cancer risk [15,16]. Thus, a comprehensive analysis which integrated all individual studies concerning this rs10877887 SNP and all cancer risk/prognosis is still required, as well as all the *let-7* family polymorphisms. And until now, a system review or a meta-analysis for the *let-7* family polymorphisms was none. These data could expand our understanding of the role of *let-7* polymorphisms in human carcinogenesis, which may provide some evidence for future research. Therefore, we systematically reviewed published data and meta-analyzed for *let-7* family polymorphisms to give a comprehensive assessment for the associations of *let-7* SNPs and cancer risks/prognosis.

## Methods

### Publication search

A literature searching was executed systematically and comprehensively by two independent investigators (B.G.W. and Q.X.), up to April 18, 2018. The databases contain PubMed, Web of Science, Embase and Chinese National Knowledge Infrastructure (CNKI) using the following key words: ‘*let-7*/pri-*let-7*’, ‘SNP/polymorphisms/variation/variant’, and ‘cancer/carcinoma/tumor/neoplasm’. The major inclusion criteria were the literatures concerning the correlation between *let-7* polymorphisms and cancer risks/prognosis. When the literature met the followings: (1) reviews or meta-analysis, (2) duplicate records, (3) study for benign disease compared with controls, (4) unrelated to cancer or *let-7* polymorphisms; it was judged as the exclusion criteria.

### Data extraction

Two authors (B.G.W. and Q.X.) extracted all the data independently, and finally reached a consensus on all the items. In the risk study, the following items were collected: first author, publication year, ethnicity, cancer type, genotyping method, source of control groups (population-based or hospital-based), total number of controls, and cases, and genotype distributions in controls and cases. In the prognosis study, the following information was extracted from the article: first author, publication year, study population, SNP names, compared genetic model, cancer type, sample size, and hazard ratio (HR) estimation. When the data in eligible articles were unavailable, we tried our best to contact the corresponding authors for original data.

### Methodology quality assessment

Quality of the selected studies was assessed according to a study regarding the method for assigning quality scores, which was mentioned in prior meta-analysis [17]. Six items were evaluated in the quality assessment scale: (1) the representativeness of the cases; (2) the source of controls; (3) the ascertainment of relevant cancers; (4) the sample size; (5) the quality control of the genotyping methods; (6) and Hardy–Weinberg equilibrium (HWE) in controls. The details see Supplementary Table S1. The quality scores of eligible studies ranged from 0 to 10. Studies with a score less than 5 and HWE disequilibrium were removed from the subsequent analyses.

### Trial sequential analysis and false-positive report probability analysis

Trial sequential analysis (TSA) was performed as described by user manual for TSA [18]. In brief, TSA software was downloaded from the website (www.ctu.dk/tsa). After adopting a level of significance of 5% for type I error and of 30% for type II error, the required information size was calculated, and TSA monitoring boundaries were built [19,20].

The false-positive report probability (FPRP) values at different prior probability levels for all significant findings were calculated as published reference studies [21–23]. Briefly, 0.2 was set as FPRP threshold and assigned a prior probability of 0.01 for an association with genotypes under investigation. A FPRP value <0.2 denoted a noteworthy association.

### Statistics

The HWE was calculated by the Chi-square test in control groups for genotype frequencies of *let-7* polymorphisms. The strength of the association between *let-7* polymorphisms and cancer susceptibility was measured by ORs and the relationship between *let-7* polymorphisms and cancer prognosis was evaluated by HRs. We calculated the between-study heterogeneity by the Cochran’s Q test and quantified by *I^2^* (a significance level of *P*<0.10). When heterogeneity did not exist, a fixed-effect model was employed [24]; otherwise, a random-effect model was used [25]. A total of five comparison models were conducted, namely heterozygote comparison (CT compared with TT), homozygote comparison (CC compared with TT), dominant model (CT + CC compared with TT), recessive model (CC compared with CT + TT), and allelic model (C compared with T).

Further, we executed stratification analyses on cancer type, source of controls (population-based and hospital-based study design), and sample size (total samples > 1000 or < 1000). The Begg’s rank correlation and the Egger’s linear regression were evaluated for the publication bias [26,27] (*P*<0.10 as reached statistically significant). All analyses were performed by STATA software, version 11.0 (STATA Corp., College Station, TX, U.S.A.).

## Results

### Characteristics of the studies

**Figure 1:**
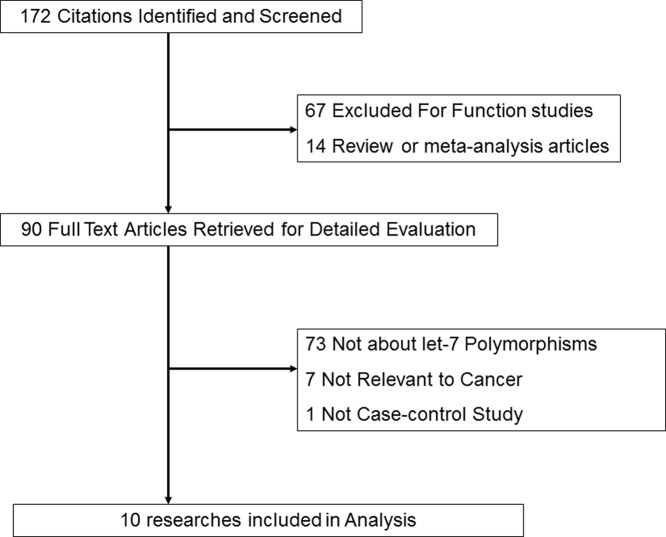
Studies identified in this meta-analysis based on the criteria for inclusion and exclusion

After duplicate literatures removed, 172 records in total were using different combinations of the major keywords. First, according to the title or abstracts screening, we excluded 81 articles (amongst them, 67 were function studies and 14 were reviews or meta-analyses). Second, after full-text reading, 81 studies were excluded (73 were not about *let-7* polymorphisms but for the *let-7* target gene polymorphisms, 7 were not associated with cancer and 1 was not case–control study). Finally, ten studies that met our inclusion criteria were included in our system review and meta-analysis, which consisted of 3837 cancer patients and 4745 controls in the risk study and 1665 cancer patients in the prognosis study (Figure 1). The characteristics of each study in the risk study were shown in Tables 1 and 2, while in the prognosis study, were presented in Table 3. This meta-analysis complied with PROSMA 2009 Checklist, and for details, see Supplementary Table S2. Amongst these ten studies, two SNPs in *let-7* family were found in risk study (*let-7i* rs10877887 and *let-7a-1/let-7f-1/let-7d* rs13293512) and three SNPs (*let-7i* rs10877887, *let-7a-1* rs10739971, and *let-7a-2* rs629367) were found in prognosis study.

**Table 1:** Characteristics of reviewed literatures for the *let-7* family polymorphisms

Number	First author	Year	Ethnicity	Cancer type	Genotyping method	Source of control groups	Sample size		miRNAs	Quality score	Citation
							Case	Control			
1	Jing Liu	2018	Asian	Cervical squamous cell carcinoma	PCR-RFLP	HB	331	358	rs10877887; rs13293512	7.5	[14]
2	ZY Sui	2016	Asian	Hepatocellular cancer	Sequencing	HB	89	95	rs10877887	6.0	[34]
3	LQ Shen	2015	Asian	Lung adenocarcinoma	Sequencing	HB	69	75	rs10877887	6.0	[35]
4	Yichao Wang	2015	Asian	Papillary thyroid carcinoma	PCR-RFLP	HB	618	562	rs10877887; rs13293512	8.5	[15]
5	Yu Zhang	2014	Asian	Oral cavity cancer	Taqman	PB	384	731	rs10877887	8.5	[16]
6	Longbiao Zhu	2014	Asian	Head and neck cancer	Sequencing	PB	497	884	rs10877887; rs13293512	8.5	[31]
7	Qian Xu	2014	Asian	Gastric cancer	PCR-RFLP; Sequencing; MassAssay	PB	579	721	rs629367; rs1143770; rs10739971; rs17276588	8.5	[29]
8	Fang Huang	2011	Asian	Hepatocellular cancer	Taqman	HB	1270	1319	rs10877887; rs13293512	7.0	[28]

HB, hospital based; PB, population based; PCR-RFLP, polymerase chain reaction-restriction fragment length polymorphism.

**Table 2:** The detailed data for the *let-7* family meta-analysis

First author	miRNAs	Year	Cancer type	Source of control groups	Sample size		Case			Control			*P* of HWE
					Case	Control	TT	TC	CC	TT	TC	CC	
Jing Liu	rs10877887	2018	Cervical squamous cell carcinoma	HB	331	358	140	131	60	169	155	34	0.860
ZY Sui	rs10877887	2016	Hepatocellular cancer	HB	89	95	25	64	64	55	40	40	0.482
LQ Shen	rs10877887	2015	Lung adenocarcinoma	HB	69	75	20	44	5	34	37	4	0.552
Yichao Wang	rs10877887	2015	Papillary thyroid carcinoma	HB	618	562	325	224	69	262	248	52	0.541
Yu Zhang	rs10877887	2014	Oral cavity cancer	PB	384	731	172	165	41	291	343	82	0.205
Fang Huang	rs10877887	2011	Hepatocellular cancer	HB	1261	1319	542	564	155	581	585	153	0.756
Longbiao Zhu	rs10877887	2014	Head and neck cancer	PB	497	884	227	213	57	361	422	101	0.179
Jing Liu	rs13293512	2018	Cervical squamous cell carcinoma	HB	331	358	97	163	71	105	186	67	0.340
Yichao Wang	rs13293512	2015	Papillary thyroid carcinoma	HB	618	562	165	333	120	158	300	104	0.066
Fang Huang	rs13293512	2011	Hepatocellular cancer	HB	1270	1291	406	611	253	427	638	226	0.642
Longbiao Zhu	rs13293512	2014	Head and neck cancer	PB	492	893	157	257	78	270	439	184	0.821

**Table 3:** The characteristics of miRNA SNPs in the prognosis study

Author name	Publication year	Study population	miRNA-SNPs	Model	Cancer type	Sample size	Outcome	HR	95% upper	95% lower	Citation
Kyung Min Shin	2016	Korea	rs1143770	CT + TT compared with CC	Non-small-cell lung cancer	761	OS	0.52	0.79	0.34	[36]
Kyung Min Shin	2016	Korea	rs629367	CC compared with AA	Non-small-cell lung cancer	761	OS	0.92	1.89	0.45	[36]
Kyung Min Shin	2016	Korea	rs10739971	GA + AA compared with GG	Non-small-cell lung cancer	761	OS	1.03	1.42	0.75	[36]
Kyung Min Shin	2016	Korea	rs17276588	GA + AA compared with GG	Non-small-cell lung cancer	761	OS	1.06	1.31	0.86	[36]
ZY Sui	2016	China	rs10877887	TT compared with CT + CC	Hepatocellular cancer	89	OS	0.68	0.94	0.52	[34]
Kaipeng Xie	2013	China	rs10877887	CT + CC compared with TT	Hepatocellular cancer	331	OS	1.23	1.58	0.96	[36]
Kaipeng Xie	2013	China	rs13293512	CT + CC compared with TT	Hepatocellular cancer	331	OS	0.93	1.22	0.71	[36]
Ying Li	2015	China	rs10739971	GA + AA compared with GG	Gastric cancer	334	OS	1.32	4.8	0.36	[37]
Qian Xu	2014	China	rs629367	CC compared with AA	Gastric cancer	150	OS	4.8	12.6	1.6	[29]

OS, overall survival.

In the risk study, all studies were matched for age; however, only seven studies were matched for sex; the other one did not need sex matching. The controls of five studies were HB, while others were PB; genotyping methods included PCR-RFLP, qPCR and sequencing. All genotypes were checked for quality control and were consistent with HWE. And according to the methodology quality assessment, the studies with a score less than 5 would be removed from the subsequent analyses. All the studies were above a score of 6.0 and recruited into the following analyses.

### Quantitative synthesis for the association of SNPs and cancer susceptibility

For the *let-7i* rs10877887 SNP, the dominate model could collect seven studies while other genetic model could collect six studies. In all the five genetic models, none was shown a significant association between *let-7i* rs10877887 SNP and overall cancer risk except the recessive model. In the recessive model, when compared with *let-7i* rs10877887 TT + TC genotype, the variant CC genotype was nearly associated with the overall cancer risk, and the *P*-value reached 0.066 (OR = 1.15; 95% confidence interval (CI) = 0.99–1.33). For the other SNP rs13293512, no association was found between the SNP and overall cancer risk (Table 4).

**Table 4:** Pooled ORs and 95% CIs of stratified meta-analysis for the risk study

Stratification	Genotype	N	OR (95% CI)	Z	*P*-value	Model	I^2^(%)
rs10877887							
All cancers							
	TC compared with TT	6	0.91 (0.76–1.09)	1.04	0.300	R	60.7
	CC compared with TT	6	1.13 (0.87–1.46)	0.93	0.351	R	54.3
	TC + CC compared with TT	7	1.10 (0.86–1.40)	0.77	0.443	R	80.9
	CC compared with TT + TC	6	1.15 (0.99–1.33)	1.84	0.066	F	45.1
	C compared with T	6	1.02 (0.89–1.16)	0.28	0.783	R	65.4
Cancer type							
Hepatocellular cancer							
	CC compared with TT + TC	2	1.85 (0.56–6.06)	1.01	0.312	R	92.9
Head and neck cancer							
	TC compared with TT	2	**0.81 (0.68–0.96)**	**2.39**	**0.017**	F	0.0
	CC compared with TT	2	0.88 (0.66–1.15)	0.95	0.341	F	0.0
	TC + CC compared with TT	2	**0.82 (0.70–0.97)**	**2.33**	**0.020**	F	0.0
	CC compared with TT + TC	2	0.98 (0.75–1.27)	0.18	0.857	F	0.0
	C compared with T	2	0.89 (0.76–1.06)	1.80	0.072	F	0.0
Source of controls							
HB							
	TC compared with TT	4	1.00 (0.76–1.31)	0.02	0.982	R	70.5
	CC compared with TT	4	1.33 (0.94–1.90)	1.59	0.111	R	57.6
	TC + CC compared with TT	5	0.82 (0.70–0.97)	1.55	0.122	R	84.2
	CC compared with TT + TC	4	1.35 (0.97–1.88)	1.76	0.079	R	56.4
	C compared with T	4	1.11 (0.92–1.33)	1.11	0.269	R	68.5
PB							
	TC compared with TT	2	**0.81 (0.68–0.96)**	**2.39**	**0.017**	F	0.0
	CC compared with TT	2	0.88 (0.66–1.15)	0.95	0.341	F	0.0
	TC + CC compared with TT	2	**0.82 (0.70–0.97)**	**2.33**	**0.020**	F	0.0
	CC compared with TT + TC	2	0.98 (0.75–1.27)	0.18	0.857	F	0.0
	C compared with T	2	0.89 (0.79–1.01)	1.30	0.072	F	0.0
Sample size							
Large							
	TC compared with TT	4	0.85 (0.72–1.01)	1.86	0.064	R	56.7
	CC compared with TT	4	1.00 (0.84–1.18)	0.02	0.985	F	0.0
	TC + CC compared with TT	4	**0.90 (0.82–1.00)**	**1.98**	**0.048**	F	50.0^a^
	CC compared with TT + TC	4	1.06 (0.90–1.24)	0.71	0.478	F	0.0
	C compared with T	4	0.96 (0.89–1.03)	1.14	0.256	F	15.9
Small							
	TC compared with TT	2	1.33 (0.69–2.56)	0.86	0.389	R	66.6
	CC compared with TT	2	**2.13 (1.36–3.35)**	**3.28**	**0.001**	F	0.0
	TC + CC compared with TT	3	**1.98(1.01–3.88)**	**1.98**	**0.048**	R	79.7
	CC compared with TT + TC	2	**2.03 (1.32–3.10)**	**3.24**	**0.001**	F	0.0
	C compared with T	2	**1.38 (1.12–1.68)**	**3.08**	**0.002**	F	0.0
rs13293512							
All cancers							
	TC compared with TT	4	1.01 (0.90–1.14)	0.18	0.861	F	0.0
	CC compared with TT	4	1.04 (0.90–1.22)	0.55	0.579	F	49.5
	TC + CC compared with TT	4	1.02 (0.91–1.14)	0.34	0.731	F	0.0
	CC compared with TT + TC	4	1.02 (0.81–1.28)	0.17	0.869	R	61.2
	C compared with T	4	1.02 (0.95–1.10)	0.52	0.603	F	34.6

a *P*_heterogeneity_ is 0.112 which is higher than 0.10, thus fixed model has been used.

**Figure 2:**
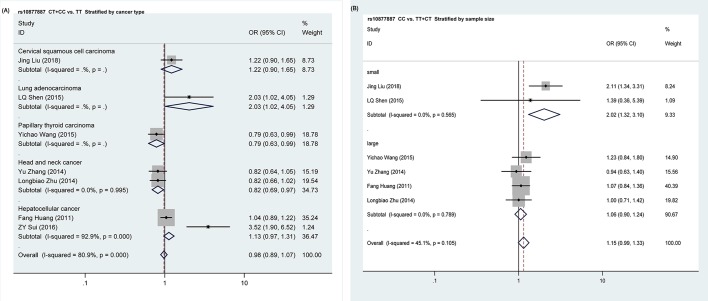
Forest plot of ORs for the association of *let-7i* rs10877887 polymorphism with cancer risks and is illustrated in subgroup analysis (**A**) Stratified by cancer type in dominate model. (**B**) Stratified by sample size in recessive model.

Furthermore, we executed stratification analysis based on different cancer types, source of controls, and sample size (Table 4). When the oral cavity cancer was divided into the head and neck cancer, the rs10877887 variant genotype was significantly associated with a decreased cancer risk in head and neck cancer (TC compared with TT: *P*=0.017; OR = 0.81; 95% CI = 0.68–0.96; TC + CC compared with TT: *P*=0.020; OR = 0.82; 95% CI = 0.70–0.97; Figure 2A). When stratified by sample size, in the small sample size subgroup, the variant genotype showed an increased significant association between rs10877887 and overall cancer risks in four genetic models (CC compared with TT: *P*=0.001; OR = 2.13; 95% CI = 1.36–3.35; TC + CC compared with TT: *P*=0.048; OR = 1.98; 95% CI = 1.01–3.88; CC compared with TT + TC: *P*=0.001; OR = 2.03; 95% CI = 1.32–3.10; C compared with T: *P*=0.002; OR = 1.38; 95% CI = 1.12–1.68; Table 4; Figure 2B). While in the large sample size subgroup, rs10877887 SNP showed a decreased risk in the dominate model (*P*=0.048; OR = 0.90; 95% CI = 0.82–1.00; Table 4).

### Quantitative synthesis for the association of SNPs and cancer prognosis

Then, we analyzed the association of *let-7* family polymorphisms and cancer overall survival. The *let-7i* rs10877887 SNP was shown to be associated with a higher risk for cancer prognosis in the dominate model (CT + CC compared with TT: *P*=0.004; HR = 1.32; 95% CI = 1.09–1.60; Table 5). The other two SNPs (*let-7a-1* rs10739971 and *let-7a-2* rs629367) were not found to be associated with cancer survival.

**Table 5:** The meta-analysis results for the association of miRNA SNPs and cancer prognosis

miRNA-SNPs	Model	Number of studies	Number of patients	HR (95% CI)	*P*	Heterogeneity (*P*)
rs10877887	CT + CC compared with TT	2	420	**1.32 (1.09–1.60)**	**0.004**	0.367
rs629367	CC compared with AA	2	911	2.01 (0.40–10.14)	0.130	0.010
rs10739971	GA + AA compared with GG	2	1095	1.05 (0.77–1.42)	0.782	0.800

### Heterogeneity

Several comparisons appeared for slight heterogeneities between studies which were shown in Table 4. We further performed sensitivity analyses to explore individual study’s influence on the pooled results by removing one study at a time from pooled analysis (Supplementary Table S3). Any significant heterogeneity was not found in any genetic models which suggested a relative reliable result.

### Publication bias

Begg’s rank correlation and Egger’s linear regression were conducted to evaluate publication bias. A slight publication bias for rs10877887 in dominate model was indicated according to the results of Begg’s test and Egger’s test (Supplementary Table S4).

### TSA and FPRP analyses

**Figure 3:**
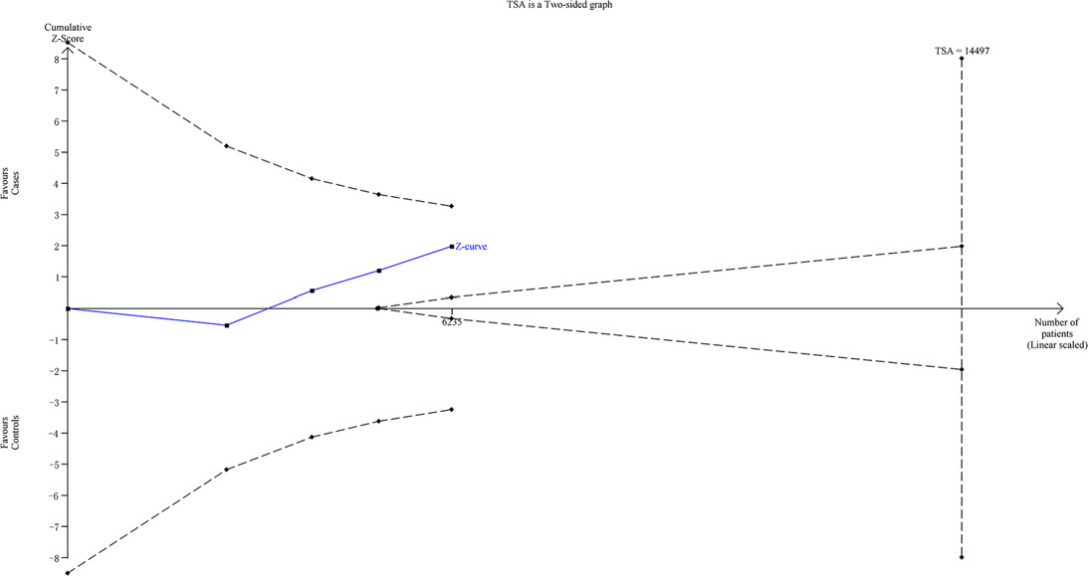
The required information size to demonstrate the relevance of *let-7i* rs10877887 polymorphism with risk of cancer in the larger sample size subgroup (dominate model)

Amongst the positive results, we found the dominate model for *let-7i* rs10877887 SNP in the larger sample size subgroup was adopted for the TSA to strengthen the robustness of our findings. According to TSA result, the required information size was 14,497 subjects to demonstrate the issue (Figure 3). Until now, the cumulative z-curve has not crossed the trial monitoring boundary before reaching the required information size, indicating that the cumulative evidence is insufficient and further trials are necessary.

Then, we calculated the FPRP values for all observed significant findings. With the assumption of a prior probability of 0.01, the FPRP values for the small sample size subgroup in the co-dominate (CC compared with TT), recessive and allelic models were all <0.20, suggesting that these significant associations were noteworthy (Table 6).

**Table 6:** FPRP values for the associations between *let-7* rs10877887 polymorphism and overall cancer risk

				Prior probability				
Variables	OR (95% CI)	*P*^a^	Power^b^	0.25	0.1	0.01	0.001	0.0001
TC compared with TT								
Head and neck cancer	0.81 (0.68–0.96)	0.017	0.666	0.071	0.187	0.716	0.962	0.996
PB	0.81 (0.68–0.96)	0.017	0.666	0.071	0.187	0.716	0.962	0.996
								
CC compared with TT								
Small sample size	2.13 (1.36–3.35)	0.001	0.922	0.003	0.010	0.097	0.520	0.916
								
TC + CC compared with TT								
Head and neck cancer	0.82 (0.70–0.97)	0.020	0.635	0.086	0.221	0.757	0.969	0.997
PB	0.82 (0.70–0.97)	0.020	0.635	0.086	0.221	0.757	0.969	0.997
Large sample size	0.90 (0.82–1.00)	0.048	0.667	0.178	0.393	0.877	0.986	0.999
Small sample size	1.98 (1.01–3.88)	0.048	0.941	0.133	0.315	0.835	0.981	0.998
								
CC compared with TT + TC								
Small sample size	2.03 (1.32–3.10)	0.001	0.899	0.003	0.010	0.099	0.526	0.918
								
C compared with T								
Small sample size	1.38 (1.12–1.68)	0.002	0.864	0.007	0.020	0.186	0.698	0.959

^a^Chi-square test was adopted to calculate the genotype frequency distributions. ^b^Statistical power was calculated using the number of observations in the subgroup and the OR and *P* values in this table.PB, source of controls is population-based

## Discussion

Concerning the history of the *let-7* family polymorphism studies, the first report began from the year of 2011. Fang Huang et al. first screened the functional SNPs from the gene region of *let-7* gene family as well as 10 kb upstream, and they selected the *let-7i* promoter rs10877887 SNP and the *let-7a-1/let-7f-1/let-7d* gene cluster promoter rs13293512 SNP as the studied polymorphism sites [28]. Almost at the same time, a few other investigators adopted a similar screening strategy and selected four SNPs as the aiming-studied SNPs (*let-7a-1* rs10739971; *let-7a-2* rs629367 and rs1143770; *let-7f-2* rs17276588) [29,30]. Although *let-7* gene family had ten gene members, only six SNPs mentioned above could be selected to study in their gene region. In our meta-analysis, only the *let-7i* rs10877887 and *let-7a-1/let-7f-1/let-7d* rs13293512 SNPs in the risk study and *let-7i* rs10877887, *let-7a-1* rs10739971, and *let-7a-2* rs629367 SNPs in the prognosis study were recruited into the pooled analysis.

The *let-7i* rs10877887 SNP was the hottest SNP in *let-7* family which all the scholars focussed on. It was located in the -286 bp region of *let-7i* gene which was the promoter region. Meanwhile, it was also located in the tail gene region of an lncRNA-linc01465. In the overall cancer risk analysis, we found that it nearly reached a statistical significance for an increased risk in recessive genetic model (Table 4). When stratified by cancer type, source of controls, and sample size, it was found that *let-7i* rs10877887 SNP variant genotype was associated with a decreased risk in dominate model in the subgroup of head and neck cancer, PB source of controls, and large sample size. While in the subgroup of small sample size, in all the genetic models, this rs10877887 SNP was associated with an increased cancer risk, except the co-dominate model (TC compared with TT). Then, we could analyze that the relative nonsignificance in the overall analysis was maybe due to the opposite results for the small and large sample size subgroups. We speculated this SNP seemed to tend to protect the cancer risk. Thus, more studies amplified sample size and multicenter studies are required in the future study to verify our findings.

The rs13293512 SNP located in -8496 bp upstream of the *let-7a-1/let-7f-1/let-7d* gene cluster which could be a promoter region for this gene cluster. For the *let-7a-1/let-7f-1/let-7d* rs13293512 SNP, only Longbiao Zhu et al. found that it was associated with head and neck cancer in the recessive genetic model [31], other three studies found no significance between this rs13293512 SNP and cancer risks. In the overall analysis, the integrated meta-analysis results also did not find this SNP had associated with cancer risk. More studies were needed to confirm this result in the future.

There is a phenomenon that even in the same kind of cancer patients with the same stage and pathological classification, the prognosis might not be the same owing to the genetic causes leading to some contributions [32]. It was accepted that the genetic polymorphisms could predict the cancer prognosis [33], and we found in this meta-analysis the *let-7i* rs10877887 SNP was associated with a higher risk for cancer prognosis in the dominate model. Due to the limited studies of the *let-7* family polymorphisms and cancer prognosis, this result need more samples to verify. And the original studies used in the meta-analysis were all hepatocellular cancer, thus this *let-7i* rs10877887 SNP maybe had the potential to be a biomarker for the specific prediction of the hepatocellular cancer prognosis.

## Advantages and limitations

To our knowledge, this is the first time to report the association between *let-7* family polymorphisms and cancer risk/prognosis. Of course, this meta-analysis still had several limitations. First, only studies written in English and Chinese were searched in our analysis, while reports in other languages or some other ongoing studies were not available. Second, the pooled sample size was relatively limited thus we could only preliminarily appraise the association of *let-7* polymorphism with currently reported types of cancers. More studies are still required to pool together to make the analysis more reliable.

## Summary and future directions

In summary, this meta-analysis suggested that the *let-7i* rs10877887 variant genotype was significantly associated with a decreased cancer risk in head and neck cancer, and the *let-7i* rs10877887 SNP was shown to be associated with a higher risk for cancer prognosis in the dominate model. Additional well-designed studies in larger samples and functional studies regarding *let-7* family SNPs are required to confirm our findings.

## Supporting information

**Table S1.** Scale for methodological quality assessment.

**Table S2.** Checklist of this meta analysis.

**Table S3.** ORs (95% CI) of sensitivity analysis.

**Supplementary Table S4:** The results of Begg’s and Egger’s test for the publication bias.

## Abbreviations

CI: confidence interval
HB: hospital based
HR: hazard ratio
HWE: Hardy–Weinberg equilibrium
NA: not available
NM: not mentioned
OR: odds ratio
PB: population based
PCR-RFLP: restriction fragment length polymorphism-polymerase chain reaction
qPCR: quantitative polymerase chain reaction
SNP: single nucleotide polymorphism
TSA: trial sequential analysis
